# The neurotrophin receptor p75 regulates gustatory axon branching and promotes innervation of the tongue during development

**DOI:** 10.1186/1749-8104-9-15

**Published:** 2014-06-24

**Authors:** Da Fei, Tao Huang, Robin F Krimm

**Affiliations:** 1 Department of Anatomical Sciences and Neurobiology, University of Louisville School of Medicine, Louisville, KY 40292, USA

**Keywords:** Taste, Gustatory, p75, Neurotrophins, TrkB

## Abstract

**Background:**

Brain-derived neurotrophic factor (BDNF) and neurotrophin-4 (NT4) regulate the survival of gustatory neurons, axon growth and branching, and innervation of taste buds during development. These actions are largely, but not completely, mediated through the tyrosine kinase receptor, TrkB. Here, we investigated the role of p75, the other major receptor for BDNF and NT4, in the development of the taste system.

**Results:**

We found that *p75*^−/−m^ice showed delayed axon outgrowth and reduced branching of gustatory axons at embryonic day (E)13.5. From E14.5 to E18.5, gustatory neurons innervated fewer papillae and completely failed to innervate the mid-region of the tongue in *p75*^−/−^mice. These early effects of the p75 mutation on gustatory axons preceded the loss of geniculate ganglion neurons starting at E14.5 and also contributed to a loss of taste buds at and after birth. Because knockouts for the *TrkB* receptor (TrkB^−/−^) do not lose as many taste buds as hybrid knockouts for its two ligands (BDNF and NT4), we asked if p75 maintains those additional taste buds in the absence of TrkB. It does not; hybrid *TrkB*^−/−^/*p75*^−/−^mice had more taste buds than *TrkB*^−/−^mice; these additional taste buds were not due to an increase in neurons or innervation.

**Conclusions:**

p75 regulates gustatory neuron axon branching and tongue innervation patterns during taste system development. This function is likely accomplished independently of BDNF, NT4, and TrkB. In addition, p75 does not support the remaining neurons or taste buds in *TrkB*^−/−^mice.

## Background

During development, axons from gustatory neurons of the geniculate ganglion grow toward and innervate specific regions of the tongue. These axons follow defined pathways, suggesting that a series of molecular cues in the environment regulate their guidance [1]. Upon arrival in the tongue, these gustatory fibers innervate specialized structures (for example, fungiform placodes) but not the surrounding epithelia. The fibers defasiculate upon entering the epithelium thereby forming a neural bud [2]. In addition to regulating the location of innervation within the tongue, molecular factors such as neurotrophins also control the number of neurons that innervate each taste bud [3].

Brain-derived neurotrophic factor (BDNF), a neurotrophin produced in developing taste papillae, regulates the survival of gustatory neurons [4,5]. Acting as a chemoattractant [6], BDNF also guides gustatory axons to their final targets [7-9]. Neurotrophin-4 (NT4) also regulates the survival of gustatory neurons, albeit earlier in development than BDNF [10,11], but has no role in axon guidance [8]. Therefore, these two neurotrophins control different aspects of taste system development. These two factors function differently primarily because of their locations and timing of expression and not due to differential signaling [12,13]. They accomplish their diverse functions by acting through the same receptors - the tyrosine kinase receptor TrkB and the pan-neurotrophic receptor p75 [14-16]. Recently, we found that neurotrophins guide a small number of TrkB-negative gustatory neurons to fungiform placodes [17]. As p75 also binds BDNF and NT4, it is possible that this receptor could mediate BDNF and NT4 function independently of TrkB. Observations that adult p75 mutant mice have fewer taste buds and geniculate ganglion neurons [18] suggest that p75 regulates the development of the taste system. However, the specific role of p75 in taste system development is difficult to predict, as p75 is known to interact with several molecular factors in a context- or system-specific manner [19-26].

The goal of the present study was to determine the role of p75 in taste system development. In particular, we examined whether p75 is required for geniculate ganglion axon guidance and neuron survival as well as the maintenance of taste buds during embryonic development by comparing tongue innervation patterns and numbers of geniculate ganglion neurons and taste buds between wild-type and mutant (*p75*^*−/−*^) mice. We found that p75 regulates geniculate ganglion axon branching and promotes innervation of the tongue but does not play a prominent role in gustatory neuron survival. Furthermore, p75 does not appear to mediate BDNF or NT4 function.

## Results

### Embryonic *p75*^*−/−*^mice show reduced geniculate ganglion axon branching and a lack of innervation in the mid-region of the tongue

**Figure 1:**
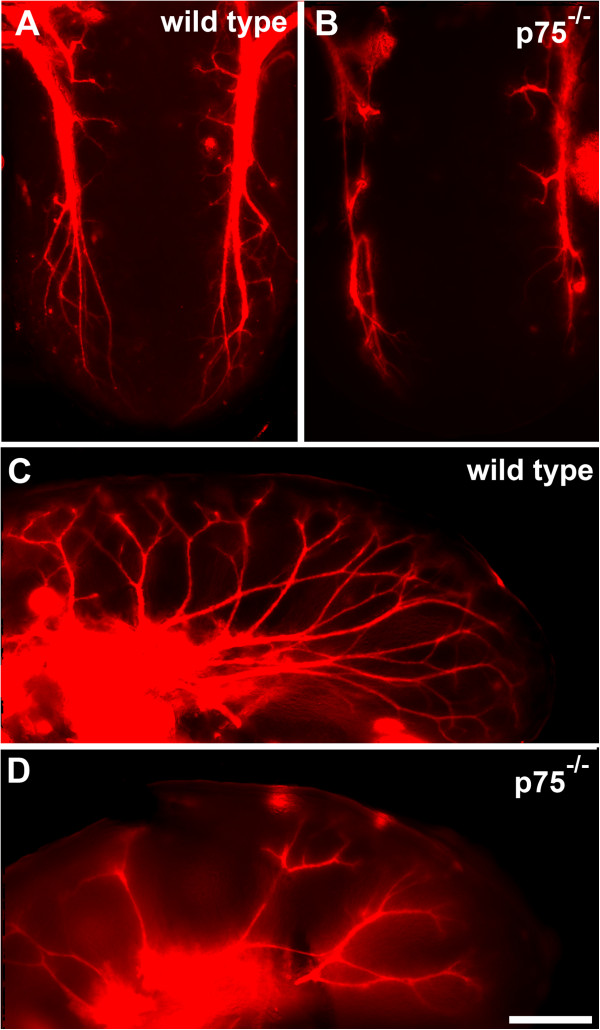
***p75***^**−/−**^**mice show reduced gustatory axon branching and innervation of the tongue mid-region on embryonic day 13.5.** DiI-labeled geniculate ganglion axons in tongues from a wild-type **(A,C)** and *p75*^−/−^**(B,D)** mouse. The dorsal view (A,B) shows reduced branching and disrupted rostral/medial progression in a *p75*^−/−^mouse. The side view **(C,D)** more clearly shows the reduction in branching. Scale bar = 250 μm.

**Figure 2:**
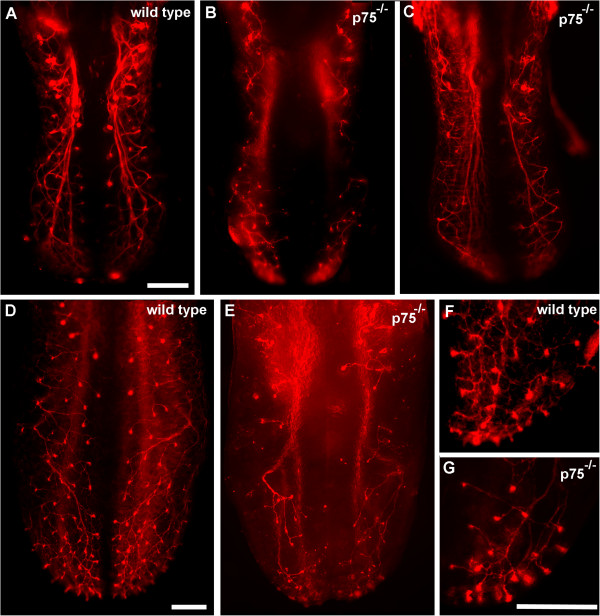
***p75***^**−/−**^**mice show reduced branching and mid-region tongue innervation on embryonic days 14.5 and 16.5.** DiI-labeled chorda tympani axons in wild-type **(A,D,F)** and *p75*^*−/−*^mice **(B,C,E,G)** on embryonic day **(E)** 14.5 **(A,B,C)** and E16.5 **(D,E,F,G)**. In *p75*^−/−^mice, chorda tympani branching was reduced, and innervation often did not reach the tongue surface as neural buds. Nerve branches were particularly absent from the tongue mid-region **(B,C,E)**. By E16.5, nerve fibers reached the tongue tip in *p75*^−/−^mice, but branching and neural bud number was reduced **(F,G)**. Scale bar = 250 μm.

We previously found that BDNF and NT4 guide a small number of neurons to fungiform placodes independently of TrkB during development [17], leading us to speculate that the p75 receptor may be important for the TrkB-independent gustatory axon guidance. To test this possibility, we labeled geniculate ganglion neurons with DiI and examined innervation of the tongue in wild-type and *p75*^*−/−*^mouse embryos. In wild-type mice on embryonic day (E)13.5, chorda tympani fiber bundles branched from the main nerve bundle toward the surface of the lateral parts of the tongue but did not yet reach the dorsal tongue surface (Figure 1A,C). Even at this early stage, *p75*^*−/−*^mice showed less axon branching than wild-type mice (Figure 1B,D). In wild-type mice on E14.5, fiber bundle branches reached the tongue surface across its entire medial-to-lateral extent, where they formed bulb-like terminations called ‘neural buds’ [2] (Figure 2A). By contrast, *p75*^*−/−*^mice showed less branching and fewer neural buds at the dorsal surface of the tongue, particularly in the mid-region, with only a few neural buds present at the tip and posterior regions of the tongue (Figure 2B-C). Because innervation of the tongue proceeds in a caudal-to-rostral direction during development [27], the presence of neural buds at the tongue tip suggests that axonal growth and targeting is not merely delayed but that branching is also reduced in *p75*^*−/−*^mice.

**Figure 3:**
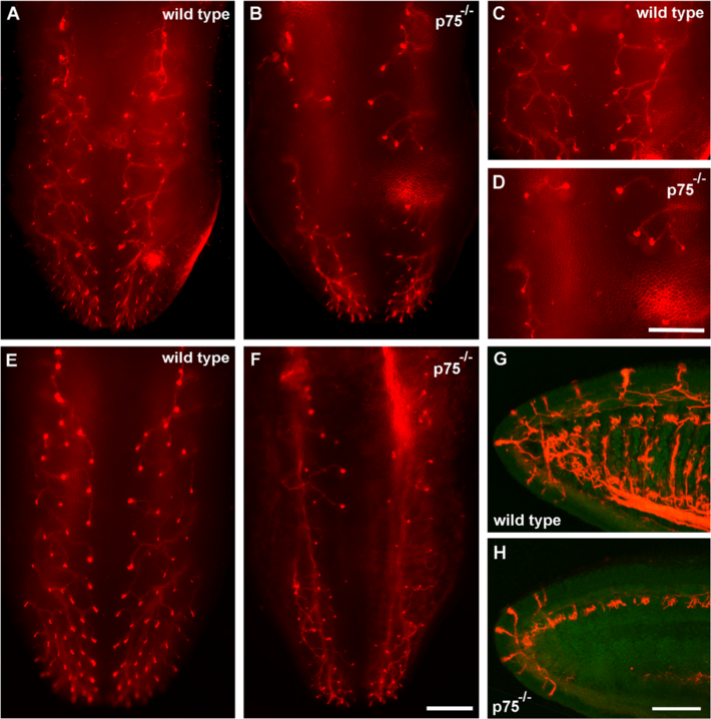
***p75***^**−/−**^**mice show reduced branching on embryonic day 18.5.** DiI-labeled chorda tympani axons in wild-type **(A,C,E)** and *p75*^*−/−*^**(B,D,F)** mice. As with the other ages, innervation was particularly sparse in the tongue mid-region of the *p75*^−/−^tongue **(D)** compared to wild-type tongue **(C)**. Sagittal sections near the tongue mid-line show that innervation reached the epithelial surface in both genotypes **(G,H)**, although *p75*^*−/−*^mice had less innervation caudal to the tongue tip. Scale bar = 500 μm for **A**, **B**, **C**, **D**, **E**, and **F**; scale bar = 200 μm for **G** and **H**.

To determine if this reduced innervation in *p75*^*−/−*^mice continues into development, we examined chorda tympani branching in wild-type and *p75*^*−/−*^mice on E16.5 and E18.5. Among wild-type mice, the pattern of axonal branching on E16.5 was similar to that on E14.5 (Figure 2D). *p75*^*−/−*^mice, however, showed an even more obvious disruption in branching, with most branches not entering the mid-region of the tongue (Figure 2E). At the tongue tip, the general pattern of branching was similar between wild-type and *p75*^*−/−*^mice, but the number of neural buds was noticeably reduced in *p75*^−/−^mice (Figure 2F,G). On E18.5, there was still an obvious deficit in innervation of the tongue mid-region in *p75*^−/−^mice compared with wild-type mice (Figure 3A-F). By sectioning the tongue and examining innervation of its dorsal surface, we confirmed that branches reached the epithelial surface in *p75*^*−/−*^mice but that innervation did not extend as far caudally as in wild-type mice (Figure 3G,H). Therefore, p75 is required for the development of normal chorda tympani branching and innervation of the mid-region of the tongue.

### Embryonic *p75*^*−/−*^mice lose geniculate ganglion neurons beginning on embryonic day 14.5

**Figure 4:**
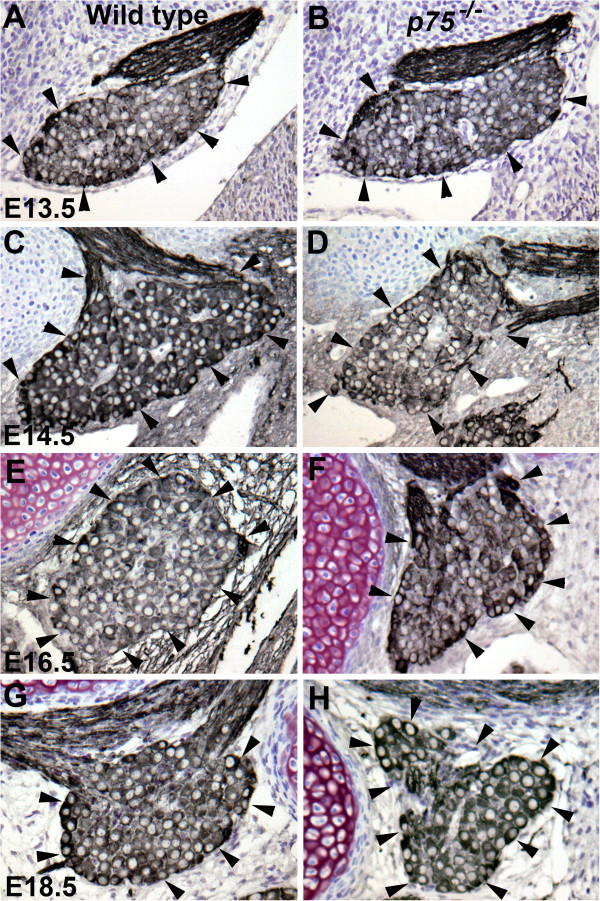
***p75***^***−/−***^**mice show less of an increase in geniculate ganglion size with embryonic age.** Anti-β-III tubulin antibody labeling of the geniculate ganglion (arrows) in wild-type **(A,C,E,G)** and *p75*^−/−^**(B,D,F,H)** mice. Beginning on embryonic day **(E)** 14.5 the ganglion appears to be slightly reduced in size in *p75*^−/−^mice compared with wild-type mice. Scale bar = 100 μm.

**Figure 5:**
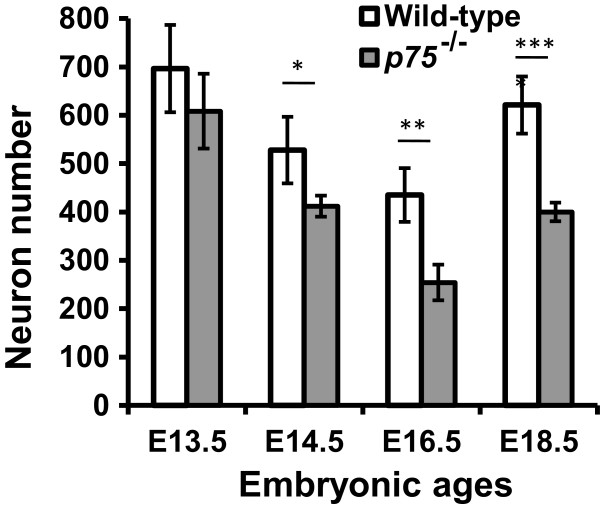
***p75***^***−/−***^**mice lose geniculate ganglion neurons starting on embryonic day 14.5.** Compared to wild-type mice, the geniculate ganglion of *p75*^*−/−*^mice contains similar numbers of neurons on embryonic day (E)13.5 but fewer neurons on E14.5 (22% decrease), E16.5 (42% decrease), and E18.5 (36% decrease). *P ≤ 0.05, **P ≤ 0.01, ***P ≤ 0.001.

Mice lacking the p75 receptor lose about 25% of geniculate ganglion neurons by adulthood [18], suggesting that our observed reduction in branching in *p75*^*−/−*^mice could be due to a loss of neurons. Geniculate ganglion neuron survival depends on both BDNF and NT4, with a loss of neurons starting at E11.5 in *Ntf4*^*−/−*^mice and at E13.5 in *Bdnf*^*−/−*^mice [5,11] and a major loss of neurons by E13.5 in *TrkB*^*−/−*^and *Bdnf*^*−/−*^*/Ntf4*^*−/−*^mice [15,17]. We reasoned that if the p75 receptor mediates geniculate ganglion neuron survival via BDNF or NT4 binding, we should see neuron loss in *p75*^*−/−*^mice by E13.5. To test this hypothesis, we examined geniculate ganglion neuron number in wild-type and *p75*^−/−^mice at E13.5 using anti-β-III tubulin antibody (TUJ-1). Geniculate ganglion neurons were easily identified by their clear nuclei and dark cytoplasm (Figure 4A,B). We found no significant neuron loss in *p75*^*−/−*^mice compared with wild-type mice at E13.5 (Figure 5). Therefore, the reduced branching in E13.5 *p75*^*−/−*^mice is not due to a loss of neurons, and p75 does not appear to act through BDNF and NT4 binding to support gustatory neuron survival during early development.

We next examined mice at later embryonic ages (E14.5, E16.5, and E18.5; Figure 4C-H) to determine when geniculate ganglion neuron loss begins in *p75*^*−/−*^mice. At all three ages, neuron number was significantly lower in *p75*^*−/−*^mice than in wild-type mice (*P* ≤ 0.05 (E14.5), *P* ≤ 0.01 (E16.5), *P* ≤ 0.001 (E18.5); Figure 5), indicating that *p75*^−/−^mice start to lose geniculate ganglion neurons at E14.5 and this loss continues throughout embryonic development. Because E14.5 is the age that gustatory nerves start to innervate peripheral targets [8], and neuron number is controlled by the amount of trophic support from these peripheral targets, this loss of neurons may be due to disrupted innervation of targets.

### *p75*^*−/−*^mice lack taste buds and taste bud innervation at birth

**Figure 6:**
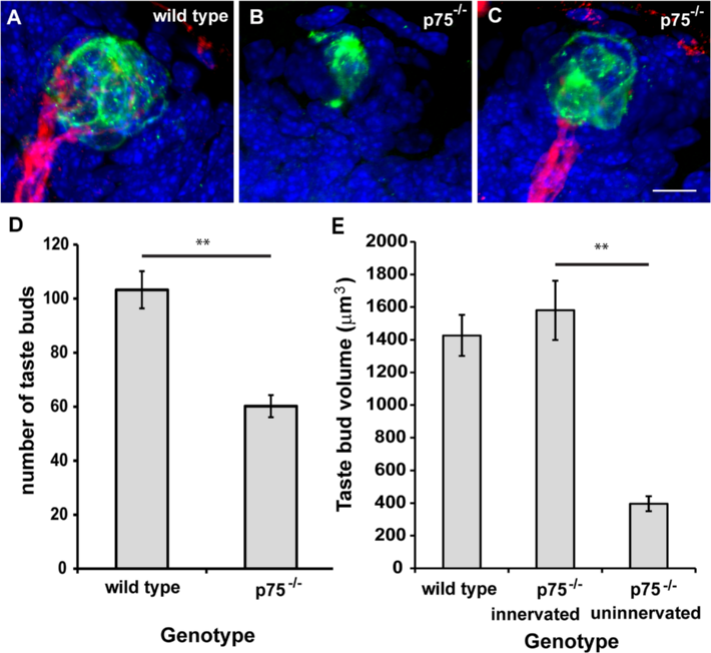
***p75***^***−/−***^**mice have fewer taste buds, and some of their existing taste buds are un-innervated. (A,B,C)** Cytokeratin 8-labeled taste buds (green), P2X3-labeled gustatory nerve fibers (red) and cell nuclei (DAPI, blue) can be seen in the lingual epithelium from a wild-type **(A)** and *p75*^−/−^**(B,C)** mouse. **(D) ***p75*^*−/−*^mice had fewer taste buds than wild-type mice. **(E) ***p75*^*−/−*^mice had similarly sized innervated taste buds but smaller un-innervated taste buds compared with wild-type mice. Scale bar = 10 μm for **A**-**C**. **P ≤ 0.01.

Neurotrophins are required for the normal development of taste buds by birth [28-30] and p75 is important for maintaining a normal number of taste buds in adulthood [18]. Given the disrupted innervation pattern observed in *p75*^−/−^mouse embryos, we hypothesized that they would show a lack of taste buds at birth. We used anti-cytokeratin-8 and anti-P2X3 to label taste buds and their innervation in wild-type and *p75*^*−/−*^mice at birth. We found that *p75*^*−/−*^mice had significantly fewer taste buds than wild-type mice (*P* < 0.001; Figure 6D). Whereas all taste buds were innervated in wild-type mice, only 80% of taste buds were innervated in *p75*^*−/−*^mice (Figure 6A-C). This finding is consistent with our earlier observation that embryonic *p75*^*−/−*^mice had un-innervated regions of the tongue, particularly the mid-region. When we measured the volume of un-innervated (Figure 6B) and innervated (Figure 6C) taste buds in *p75*^−/−^mice, we found that innervated taste buds were significantly larger than un-innervated taste buds (Figure 6E, *P* ≤ 0.01). Therefore, p75 receptor mutation disrupted innervation of the tongue during development, reducing both the number and volume of taste buds.

It is well established that innervation is required to support the existence of taste buds during postnatal development [31,32], suggesting that un-innervated taste buds could be lost at later ages. Therefore, we quantified taste bud number in wild-type and *p75*^*−/−*^mice at postnatal day (P)10. Whereas wild-type mice had 116 ± 7 taste buds, *p75*^*−/−*^mice had only 75 ± 2 taste buds. However, no additional taste buds were lost in *p75*^*−/−*^mice at P10 (35%) compared with birth (42%), indicating that un-innervated taste buds may continue to be supported postnatally.

### p75 is not responsible for supporting the existence of taste buds in *TrkB*^*−/−*^mice

We previously showed that *TrkB*^−/−^mice possess more taste buds than hybrid *Bdnf*^−/−^/*Nft4*^−/−^mice [17], perhaps because BDNF or NT4 function through other types of receptor in the absence of TrkB. One likely possibility is the p75 receptor, as we showed earlier p75 is required for normal innervation of the tongue surface. If BDNF and NT4 function via p75 to support the additional taste buds in *TrkB*^−/−^mice, the number of taste buds should be reduced in hybrid *TrkB*^−/−^/*p75*^*−/−*^mice. To test this possibility, we quantified taste buds in *TrkB*^−/−^/*p75*^*−/−*^, *TrkB*^−/−^, *p75*^*−/−*^, and wild-type mice at E13.5. Surprisingly, we observed an increase in taste bud number in *TrkB*^*−/−*^/*p75*^*−/−*^mice (53 ± 11) compared with *TrkB*^−/−^mice (31 ± 8; *P* < 0.01). The number of taste buds in *TrkB*^*−/−*^/*p75*^*−/−*^mice was similar to that in *p75*^−/−^mice (60 ± 9) but still significantly less than in wild-type mice (103 ± 14; *P* <0.001). Therefore, p75 does not appear to support the remaining taste buds in *TrkB*^−/−^mice.

**Figure 7:**
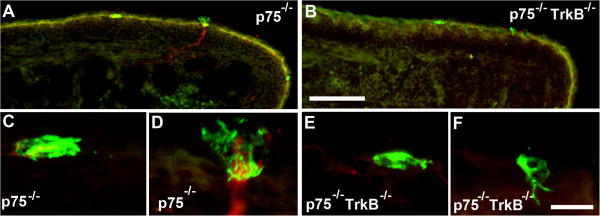
**Hybrid *TrkB***^**−/−**^**/*p75***^**−/−**^**mice have many un-innervated taste buds at birth.** Keratin 8-labeled (green) and P2X3-labeled (red) tongue sections from **(A)** a *p75*^*−/−*^and **(B)** a *TrkB*^*−/−*^*/p75*^*−/−*^mouse. Whereas the *p75*^−/−^mouse had both innervated **(D)** and un-innervated **(C)** taste buds, the *TrkB*^−/−^/*p75*^−/−^mouse had mostly un-innervated taste buds **(E,F)**. Scale bar = 100 μm for **A** and **B**; scale bar = 10 μm for **C-F**.

Interestingly, in other systems, p75 causes the death of some neurons in the absence of Trk receptors [33,34]. Therefore, we speculated that this might also be true for gustatory neurons, with rescued gustatory neurons supporting the additional taste buds in *TrkB*^−/−^/*p75*^*−/−*^mice. To test this idea, we quantified geniculate ganglion neuron number in *TrkB*^−/−^/*p75*^*−/−*^mice on E13.5, at which time a deficit in neuron number is observed in *TrkB*^−/−^mice. We found that *TrkB*^*−/−*^/*p75*^*−/−*^mice also lacked geniculate ganglion neurons at E13.5, with no significant difference in the number of remaining neurons between *TrkB*^*−/−*^ (97 ± 18) and *TrkB*^*−/−*^/*p75*^*−/−*^mice (107 ± 10). Therefore, the deletion of p75 does not appear to increase the number of taste buds in *TrkB*^−/−^mice by rescuing neurons, which is consistent with our observation that the additional taste buds in *TrkB*^*−/−*^/*p75*^*−/−*^mice did not appear to be innervated (Figure 7A-F). These results indicate that p75 may promote apoptosis in the taste bud in the absence of innervation.

## Discussion

Our goal was to determine the role of the neurotrophin receptor p75 in taste system development. In particular, because we previously found that a small subpopulation of TrkB-negative gustatory axons require BDNF to navigate to taste buds through an unknown receptor [17], we tested whether p75 could be this receptor and regulate axon guidance and innervation of targets in a manner similar to BDNF. We found that deletion of *p75* disrupted innervation of the tongue surface during development. Specifically, peripheral axons branched less and did not innervate the mid-region of the tongue in embryonic *p75*^−/−^mice. As this pattern of innervation was unlike that previously observed in BDNF mutant mice [8], p75 probably does not mediate the role of BDNF in axon guidance. We also found that geniculate ganglion neurons were lost after alterations in axonal branching in p75^-/-^ mice, suggesting that disrupted innervation influences neuron survival in *p75*^−/−^mice, and not that neuron death disrupts innervation patterns. The reduced innervation to the tongue surface resulted in taste bud loss, with only 80% of remaining taste buds being innervated in *p75*^*−/−*^mice.

In *p75*^*−/−*^mouse embryos, the chorda tympani nerve branched less and failed to innervate the mid-region of the tongue. Because innervation progresses in a caudal/lateral-to-medial/rostral direction as nerve fibers grow into the tongue [2,35-37], this lack of innervation could be due to delayed or inhibited nerve outgrowth. This possibility is consistent with the finding that blocking p75 function *in vitro* reduces BDNF-stimulated outgrowth of geniculate ganglion neurons [38]. In the whisker pad, the absence of p75 delays trigeminal innervation, decreases axon branching, and is associated with delayed glial development [39]. Unlike trigeminal axons, however, which eventually innervate their targets, *p75*^−/−^mice show a consistent deficit in innervation of the tongue through E18.5, leading to a permanent reduction in the number of taste buds [18].

At all ages observed, most gustatory nerve endings were missing in the middle and caudal areas of the tongue in *p75*^−/−^mice. This pattern of innervation is different from that of *Bdnf*^−/−^mice, in which branching is increased and fibers extend to all regions of the tongue but fail to innervate fungiform papillae [8]. Thus, although p75 is a receptor for BDNF, it does not appear to mediate the role of BDNF in axon guidance, although it could partially mediate the role of BDNF in axon outgrowth [38]. Rather, it is more likely that p75 functions independently of TrkB ligands and instead interacts with a different factor to influence axon guidance. p75 is known to interact with several different guidance molecules, including ephrins [40]. Also, p75 modulates activity of the chemorepellent semaphorin3A (Sema3A). Dorsal root ganglion neurons in *p75*^*−/−*^mice are hypersensitive to Sema3A [41], and Sema3A acts as a chemorepellent against geniculate ganglion axons *in vitro*[37], with its repellent effect being neurotrophin-dependent [42]. Sema3A is expressed in the tongue epithelium early in development, and its expression slowly decreases in a lateral-to-medial direction as the tongue becomes innervated [43], thereby preventing premature innervation. Furthermore, Sema3A mutant mice show increased innervation of the tongue mid-line [37]. Thus, our finding of decreased innervation of the mid-region of the tongue in *p75*^*−/−*^mice is consistent with a hypersensitivity to Sema3A. In general, however, interactions of the p75 receptor with many different signaling factors could be important for axon guidance and targeting.

We found that *p75*^*−/−*^mice lose geniculate neurons by E14.5 and that this loss continues through E18.5. Because this loss occurs after neurons become dependent on BDNF and NT4 [5,11,44] it is not likely that p75 functions by mediating the effects of neurotrophins on neuron survival, at least not initially. Normally, gustatory nerves reach the tongue epithelium and innervate fungiform placodes by E14.5 [2,8]. In *p75*^−/−^mouse embryos, fewer fungiform papillae were innervated, indicating that neurons failed to reach their targets. Therefore, these neurons could die starting at E14.5 due to a lack of survival factors received from the targets. Consistent with this idea, we observed only a 30% loss in geniculate ganglion neuron number at E14.5, and no neuron loss at E13.5 even though axon branching was already disrupted. Given that alterations in innervation patterns were more severe and occurred earlier than neuron loss, it is more likely that disrupted innervation caused neuron loss rather than *vice versa*. Neuron loss in other sensory ganglia could also be due to failed targeting [39,45], which could explain why sensory neurons tend to be lost in *p75*^−/−^mice [46,47] whereas other neuron populations (for example, sympathetic neurons) are increased in the absence of p75 [33].

The p75 receptor can promote neuronal apoptosis in the absence of Trk receptors [33,34,48]; for example, nerve growth factor causes the death of retinal neurons that express p75 but not TrkA [48]. Also, BDNF activates p75 to induce apoptosis of sympathetic neurons [33], which do not express TrkB. Based on these findings, we examined whether p75 causes the death of geniculate ganglion neurons in the absence of TrkB and whether the additional removal of p75 can rescue these neurons. Surprisingly, *TrkB*^*−/−*^*/p75*^*−/−*^mice had similar numbers of neurons as *TrkB*^*−/−*^mice. This finding suggests that p75 plays a different role in the development of the taste system compared with other systems [33,48,49], perhaps due to differences among systems in Trk receptor function. In the retina [48], sympathetic ganglia [33], and dorsal root ganglia [49], p75 induces apoptosis specifically in the absence of TrkA. Thus, TrkB may regulate cell death differently than TrkA [49], and the actions of p75 may differ in the absence of these two receptor types. Regardless of the underlying reason, our results indicate that p75 does not cause neuronal apoptosis when TrkB is absent during taste system development.

Because taste buds require support from nerve fibers to retain their normal numbers and size [50-53], taste buds are lost in the absence of neurotrophins [28-30,54]. Likewise, since innervation to the tongue was disrupted, p75 mice also had a loss of taste buds. In all cases with neurotrophin knockout mice some un-innervated taste buds, as defined by keratin 8 labeling, remain [15,54]. These taste bud remnants also remain following nerve section [50,55]. Consistent with this literature we found un-innervated taste buds that remained in p75^−/−^mice. Because nerve section results in a greater loss of taste buds during development than it does in adulthood [53,56,57], we thought that perhaps these additional taste buds would be lost postnatally, but that was not the case; they still remained in P10 *p75*^−/−^mice. It seems likely that these smaller remaining un-innervated collections of K8-positive cells simply do not require innervation to be maintained.

Not all neurotrophin knockout and receptor mice lose the same number of taste buds; for example, *TrkB*^−/−^mice possess more taste buds than hybrid *Bdnf*^−/−^/*Nt4*^−/−^mice at birth [15,17,58]. The reason for this is unclear but we speculated that the additional taste buds might be supported by BDNF or NT4 functioning via p75. However, mice with both TrkB and p75 mutations possessed more taste buds than *TrkB*^−/−^mice, suggesting that p75 is not required for the additional taste buds in *TrkB*^−/−^mice. Although this increase in number of taste buds was surprising, p75 has been found to cause the death of neurons in the absence of Trk expression [33,34,48]. When we investigated whether the increase in taste bud number in *TrkB*^−/−^/*p75*^−/−^mice might be supported by a larger number of neurons, we found that geniculate ganglion neuron number was unaffected by mutation of both TrkB and p75, and few of the taste buds in *TrkB*^−/−^/*p75*^−/−^mice appeared to be innervated. Therefore, p75 does not cause the death of neurons in the absence of Trk signaling in the taste system, and the additional taste buds in *TrkB*^−/−^/*p75*^−/−^mice relative to *TrkB*^−/−^mice are not rescued by a greater number of neurons. Together, these findings suggest that, although p75 regulates innervation of the tongue surface, it does not mediate the role of BDNF in axon guidance or the role of TrkB ligands in neuron survival.

## Conclusions

The pan-neurotrophic factor receptor, p75, regulates gustatory innervation patterns and branching of gustatory fibers in the tongue. Although gustatory neurons are lost in *p75*^−/−^mice this appears to be downstream of the innervation deficits, and is not likely due to a direct role of p75 in mediating BDNF- and/or NT4-regulated survival. The taste buds previously shown to be remaining in *TrkB*^−/−^mice, but not *Bdnf*^*−/−*^*/Nt4*^−/−^mice, are not maintained by p75 and leave open the possibility that the two factors function through an additional receptor other than TrkB and p75 to regulate gustatory development.

## Methods

### Animals

Heterozygous *TrkB*^*+/−*^ (stock no. 002544; Jackson Laboratories, Bar Harbor, ME, USA) and *p75*^*+/−*^ (stock no. 002213; Jackson Laboratories) mice were bred to produce *p75*^*−/−*^and *TrkB*^*−/−*^*/p75*^*−/−*^mice. The day that a sperm plug was positively identified was designated E0.5. Mice were genotyped using polymerase chain reaction. All procedures were approved by the University of Louisville Institutional Animal Care and Use Committee committee and are in accordance with the guidelines of the US Public Health Service Policy on Humane Care and Use of Laboratory Animals and the National Institutes of Health Guide for the Care and Use of Laboratory Animals.

### Quantification of geniculate ganglion neurons

E13.5 (*p75*^*−/−*^n = 3*, TrkB*^*−/−*^*/p75*^*−/−*^n = 2, and wild-type *n* = 3), E14.5 (*p75*^*−/−*^n = 3*,* and wild-type *n* = 3), E16.5 (*p75*^*−/−*^n = 3*,* and wild-type *n* = 3), and E18.5 (*p75*^*−/−*^n = 3*,* and wild-type *n* = 3) embryos were transcardially perfused with ice-cold 4% phosphate-buffered paraformaldehyde (PFA) and post-fixed overnight in 4% PFA. Following fixation, embryo heads were dissected, transferred to 70% ethanol, and processed for paraffin embedding. Geniculate ganglion neurons were visualized using TUJ-1 antibody as previously described [5]. Briefly, serial sections (5 μm) of paraffin-embedded embryos were collected on SuperFrost Plus slides (Fisher Scientific, Waltham, MA, USA). Paraffin was then removed by immersion in Citrisolv (Fisher Scientific) overnight. Following rehydration and endogenous peroxidase blocking, slides were treated for antigen retrieval in citrate buffer (0.1 M citric acid and 0.1 M sodium citrate in dH_2_O; pH 6). Sections were washed in PBS, blocked for 1 hour in blocking solution (5% goat serum and 0.25% Triton X-100 in PBS), and incubated overnight in blocking solution containing mouse TUJ-1 antibody (1:500; #MMS-435P, Covance, Princeton, NJ, USA). The next day, sections were washed and incubated for 1.5 hours in blocking solution containing biotinylated anti-mouse secondary antibody (1:250; #BA-2000, Vector Laboratories, Burlingame, CA, USA) and visualized using an ABC diaminobenzidine reaction kit (#PK-6200; Vector Laboratories).

TUJ-1 immunostaining was used to quantify geniculate ganglion neurons in sections in which nuclei were visible. Neurons were counted in six sections per ganglion. The area of the geniculate ganglion in each of these six sections was measured, multiplied by section thickness, and summed to provide the total volume of quantified sections. The volume of the entire geniculate ganglion was measured in the same way, using every section containing the geniculate ganglion. The total number of neurons in the ganglion was estimated by multiplying the number of neurons per volume of the quantified sections by total ganglion volume. This number was multiplied by a correction factor to compensate for the presence of individual nuclei appearing in multiple sections [59]. This correction factor was calculated as N = n × [T/(T × D)], where N is the estimated total number of neurons, n is the number of neurons counted, T is the section thickness, and D is the average diameter of nuclei [5].

### Quantification of taste buds

Mice at day of birth (*p75*^*−/−*^n = 5*, TrkB*^*−/−*^*/p75*^*−/−*^n = 3, and wild-type *n* = 4) and P10 (*p75*^*−/−*^n = 3 and wild-type *n* = 3) were anesthetized and transcardially perfused with ice-cold 4% PFA. The front of the tongue containing the fungiform field was separated and post-fixed in 4% PFA for 2 hours. Tongues were then placed in 30% sucrose overnight. The next day, tongues were embedded in OCT medium (#4583; Sakura Finetek USA, Inc., Torrance, CA, USA). Serial sagittal sections (25 μm) were collected onto SuperFrost Plus slides (Fisher Scientific). For antigen retrieval, sections were heat-dried overnight, rehydrated, placed into citrate buffer (pH 6.0), heated for 15 minutes in a boiling water bath, and incubated for 10 minutes at room temperature.

Primary rat anti-cytokeratin 8 antibody (1:50; Developmental Studies Hybridoma Bank, Iowa City, IA, USA) and rabbit anti-P2X3 antibody (#AB5895; 1:500, Millipore, Billerica, MA, USA) were used to label gustatory nerves and taste buds. Cytokeratin 8 is a marker of simple epithelia that labels most columnar taste cells [60,61], and P2X3 is an ATP channel that is required for taste function [62] and expressed in most geniculate ganglion neurons innervating the tongue [63]. Secondary anti-rat Alexa 488 (green) and anti-rabbit Alexa 555 (red) antibodies (1:500, Molecular Probes, Grand Island, NY, USA) were used to visualize cytokeratin 8- and P2X3-containing nerve fibers, respectively. Sections were examined in order, and individual taste buds were followed across sections so that each taste bud was only counted once.

To assess innervation of taste buds, confocal stacks of optical sections with a Z-step of 0.5 μm were imaged for three to five taste buds from each mouse (*p75*^*−/−*^n = 5*,* and wild-type *n* = 4) and analyzed using ImageJ software (http://rsbweb.nih.gov/ij/). The area of the taste bud in each optical section was measured, and these areas were summed and multiplied by section thickness to calculate taste bud volume. The area of P2X3-positive staining within outlined taste buds was also measured in each optical section, and these areas were summed and multiplied by section thickness (0.5 μm) to calculate the volume of P2X3 labeling within taste buds. The percentage of taste bud volume occupied by innervation was calculated by dividing the volume of P2X3 labeling by the volume of cytokeratin 8 labeling.

### DiI-labeling of the geniculate ganglia

E14.5 (*p75*^*−/−*^n = 3, wild-type *n* = 3), E16.5 (*p75*^*−/−*^n = 6, wild-type *n* = 5), and E18.5 (*p75*^*−/−*^n = 4, wild-type *n* = 6) mice were anesthetized and transcardially perfused in ice-cold 4% PFA. E13.5 mice were immersion-fixed. DiI labeling was performed as previously described [64]. Briefly, brains were removed and DiI crystals were placed on the geniculate ganglion and facial nerve. Embryos were incubated at 37°C for 2 to 8 weeks depending on their age. The tongue was then dissected, examined, and photographed using a fluorescent dissecting microscope (Leica Microsystems, Wetzlar, Germany) equipped with a camera (QImaging, 19535 56th Avenue, Suite 101 Surrey, BC ).

### Data analysis

The total number of neurons was compared between genotypes on E13.5, E14.5, E16.5, and E18.5 using two-way analysis of variance. Geniculate ganglion neuron number and taste bud number and volume were compared using one-way analysis of variance. Statistical significance was set at *P* < 0.05. Data are reported as mean ± standard error of the mean in the text and figures.

## Abbreviations

BDNF: brain-derived neurotrophic factor; DiI: (2*Z*)-2-[(*E*)-3-(3,3-dimethyl-1-octadecylindol-1-ium-2-yl)prop-2-enylidene]-3,3-dimethyl-1-octadecylindole perchlorate; E: embryonic day; NT4: neurotrophin-4; P: postnatal day; P2X3: purinoceptor 3; p75: pan-neurotrophin receptor; PBS: phosphate-buffered saline; PFA: paraformaldehyde; Sema3A: semaphorin 3A; TrkA: tropomyosin related kinase A; TrkB: tropomyosin related kinase B; TUJ1: anti-β-III tubulin antibody.

## Competing interests

The authors declare that they have no competing interests.

## Authors’ contributions

DF carried out most of the studies and drafted the initial manuscript. TH carried out the E13.5 Di-labeling study. RFK conceived of the study, participated in its design, constructed most of the final figures, determined the percentage of taste buds that were innervated in p75^−/−m^ice and drafted the final manuscript. All authors read and approved the final manuscript.
